# Empagliflozin demonstrates cytotoxicity and synergy with tamoxifen in ER-positive breast cancer cells: anti-proliferative and anti-survival effects

**DOI:** 10.1007/s00210-024-03316-z

**Published:** 2024-07-27

**Authors:** Ahmad Karzoon, Mükerrem Betül Yerer, Ahmet Cumaoğlu

**Affiliations:** 1 Department of Pharmacology, Faculty of Medicine, Erciyes University, Kayseri, Türkiye; 2 Drug Application and Research Center (ERFARMA), Department of Pharmacology, Faculty of Pharmacy, Erciyes University, Kayseri, Türkiye; 3 Department of Biochemistry, Faculty of Pharmacy, Erciyes University, Kayseri, Türkiye

**Keywords:** Empagliflozin, ERα + breast cancer, FOXO3a, PGC-1α, Akt, p70S6K1

## Abstract

Accumulating evidence suggests that sodium–glucose cotransporter 2 (SGLT2) inhibitors may be effective at eliminating tumor cells. While empagliflozin exhibits nearly the highest selectivity for SGLT2 over SGLT1, its specific impact alone and in combination with tamoxifen remains largely unexplored in estrogen receptor α-positive (ERα +) breast cancer. This study investigated the anticancer effects of empagliflozin and its potential synergy with tamoxifen in MCF-7 breast cancer cells. The individual and combined cytotoxic effects of empagliflozin and tamoxifen were assessed using the xCELLigence system. The activities of AMP-activated protein kinase α (AMPKα), p38 mitogen-activated protein kinase (p38 MAPKα), p70-S6 kinase 1 (p70S6K1), and protein kinase B (Akt) were assessed using Western blotting. The gene expression levels of peroxisome proliferator-activated receptor-gamma coactivator-1α (PGC-1α) and Forkhead box O3a (FOXO3a) were assessed via qPCR. Our results revealed time- and concentration-dependent cytotoxic effects of empagliflozin and tamoxifen whether administered separately or in combination. While tamoxifen exhibits potency with an IC_50_ value of 17 μM, approximately ten times greater than that of empagliflozin (IC_50_ = 177 μM), synergistic effects are observed when the concentrations of the two agents approach their respective IC_50_ values. Additionally, empagliflozin significantly increases AMPKα activity while concurrently inhibiting Akt, p70S6K1, and p38 MAPKα, and these effects are significantly enhanced when empagliflozin is combined with tamoxifen. Moreover, empagliflozin modulates the gene expression, downregulating PGC-1α while upregulating FOXO3a. Empagliflozin exerts anti-proliferative and anti-survival effects by inhibiting mTOR, Akt, and PGC-1α, and it exhibits synergy with tamoxifen in MCF-7 breast cancer cells.

**Graphical Abstract:**

Proposed anticancer mechanism of empagliflozin in MCF-7 breast cancer cells.

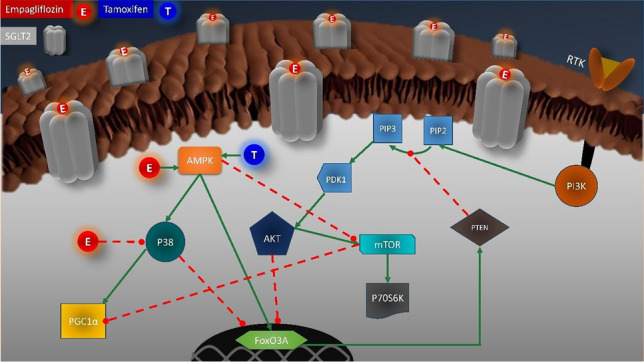

**Supplementary Information:**

The online version contains supplementary material available at 10.1007/s00210-024-03316-z.

## Introduction

The global prevalence of breast cancer and its associated mortality represent significant healthcare challenges. In 2022, there were more than 2.3 million new cases and 665,684 deaths attributed to breast cancer (Bray et al. 2024). Should prevailing patterns persist, projections indicate a notable escalation in the burden of breast cancer, with estimates surpassing 3 million new cases annually and a mortality rate exceeding 1 million deaths per year by 2040, primarily due to population expansion and demographic aging (Arnold et al. 2022).

Within the spectrum of molecular subtypes present in breast cancer, estrogen receptor α-positive (ERα +) breast cancer is the predominant category, accounting for 70% of all instances of breast malignancies (Clarke et al. 2001). Tamoxifen is a cornerstone adjuvant therapy for ERα + breast cancer. Unfortunately, approximately 30% of ERα + breast cancer cases do not respond to tamoxifen treatment and many tumors that initially respond eventually develop resistance (Clarke et al. 2001).

It is widely recognized that cancer cells exhibit significantly accelerated growth and proliferation compared to normal human cells (Hanahan and Weinberg 2000). Nonetheless, the tumor microenvironment presents a challenge with its limited nutrient availability, prompting cancer cells to undergo metabolic reprogramming to acquire substantial amounts of energy and materials (Hanahan and Weinberg 2011). Among the various metabolic pathways, glucose metabolism is particularly important in cancer cell biology.

Given the importance of metabolic reprogramming, previous endeavors to inhibit glucose transporter proteins (GLUTs) were deemed impractical as these transporters are essential for maintaining the biological functions of healthy cells. Intriguingly, SGLT2s have been found to be overexpressed in a range of cancer cell types, including hepatocellular carcinoma, pancreatic, prostate, colorectal, lung, and breast cancers; and tumors of the brain, head, and neck (Billger et al. 2019; Ishikawa et al. 2001; Kaji et al. 2018; Koepsell 2017; Komatsu et al. 2020; Perry and Shulman 2020; Wright et al. 2011; Yamamoto et al. 2021; Zhang et al. 2019; Zhou et al. 2020). Accumulating evidence suggests that SGLT2 inhibitors may be effective at eliminating tumor cells.

Studies have indicated that breast cancer cells exposed to low micromolar concentrations of tamoxifen show increased reliance on glucose metabolism. Moreover, inhibiting this process pharmacologically has been found to be synergistically lethal with tamoxifen (Daurio et al. 2016). While empagliflozin exhibits nearly the highest selectivity for SGLT2 over SGLT1 (2680:1) among other SGLT2 inhibitors, its specific impact alone and in combination with tamoxifen remains largely unexplored in estrogen receptor α-positive breast cancer.

Our hypothesis suggests that empagliflozin enhances intracellular adenosine monophosphate (AMP) levels, thereby activating AMP-activated protein kinase α (AMPKα). AMPKα serves as a crucial regulator, inhibiting mammalian target of rapamycin complex 1 (mTORC1) and its substrate, p70-S6 kinase 1 (p70S6K1) (Chaube et al. 2015). However, the AMPKα-p38 mitogen-activated protein kinase α (p38 MAPKα)-peroxisome proliferator-activated receptor-gamma coactivator-1α (PGC-1α) axis is implicated in promoting cancer cell survival under glucose-limiting conditions (Chaube et al. 2015). Interestingly, studies have shown that empagliflozin inhibits p38 MAPKα in the livers of mice with hepatocellular carcinoma (Abdelhamid et al. 2022). Another downstream target of AMPKα is Forkhead box O3a (FOXO3a). FOXO3a enhances phosphatase and tensin homolog (PTEN) transcription to counteract phosphatidylinositol-3 kinase (PI3K)/ protein kinase B (Akt) pathway hyperactivity (Nasimian et al. 2020; Sajjadi et al. 2021). In the context of endocrine therapy resistance, multiple factors have been identified that suppress AMPKα (Casimiro et al. 2017; Lopez-Mejia et al. 2017; Yi et al. 2020). In ER + breast cancer, the interaction between the PI3K-Akt-mTOR and estrogen receptor α pathways drives resistance to endocrine therapy (Cassinelli et al. 2013). Furthermore, high expression levels of p70S6K are associated with resistance to endocrine therapy and poor prognosis (Kim et al. 2011).

This study aimed to investigate the anticancer effects of empagliflozin and its potential synergistic effects with tamoxifen in MCF-7 breast cancer cells. With a focus on AMPKα activity, we investigated the effects of empagliflozin alone and in combination with tamoxifen on downstream pathways implicated in anticancer mechanisms that may contribute to the attenuation of tamoxifen resistance. These pathways include PGC-1α, p38 MAPKα, p70S6K1, FOXO3a, and Akt.

## Materials and methods

### Reagents

Empagliflozin (#BD289522) was purchased from BLD Pharm (Shanghai, China). Tamoxifen citrate (#10051) was purchased from Chemische Fabrik Berg GmbH (Bitterfeld-Wolfen, Germany). Dulbecco's phosphate buffered saline (#D1408), fetal bovine serum (#F9665), penicillin–streptomycin (#P4333), trypsin–EDTA (#T4049), and Dulbecco’s Modified Eagle’s Medium (#D5546) were purchased from Sigma-Aldrich (St. Louis. MO, USA). All the other chemicals utilized were of analytical grade.

### Cell culture

MCF-7 human breast cancer cells were generously provided by ERFARMA (Erciyes University, Türkiye). The cells were cultured in Dulbecco’s Modified Eagle’s Medium (DMEM) supplemented with 10% fetal bovine serum (FBS) and 1% penicillin/streptomycin and maintained at 37°C in a humidified atmosphere containing 5% CO2. The cells were cultured as a monolayer in 75 cm2 flasks. Upon reaching 80% confluence, the cells were subcultured with 0.25% Trypsin-0.53 mM EDTA solution. The resulting MCF-7 cell pellet was seeded onto 6-well plates or e-plates for subsequent analysis.

### Cytotoxicity assay and real-time cell monitoring

The optimal seeding concentration of the MCF-7 cell line was initially determined. Subsequently, 15,000 cells were seeded in DMEM culture medium supplemented with 10% FBS and 1% penicillin/streptomycin for a 24-h incubation period in 16-well e-plates (Agilent Technologies, California, USA). Growth curves were then automatically recorded in real-time every 10 min using the xCELLigence system (ACEA Biosciences Inc., California, USA). During the logarithmic growth phase, control cells were administered medium containing the vehicle, and the final concentration of dimethyl sulfoxide (DMSO) did not exceed 0.5%. Moreover, the test cells were exposed to various concentrations of empagliflozin (75 μM, 150 μM, 300 μM, or 600 μM), tamoxifen (5 μM, 7.5 μM, 10 μM, 15 μM, 20 μM, 30 μM, or 40 μM), or combinations of both (empagliflozin/tamoxifen: 180 μM/15 μM, 180 μM/17.5 μM, 180 μM/20 μM, 90 μM/9 μM, 140 μM/14 μM, or 160 μM/16 μM). The stock solutions of empagliflozin and tamoxifen were prepared according to the highest concentrations used in each experiment. For instance, to prepare concentrations of 600 μM for empagliflozin and 40 μM for tamoxifen, empagliflozin and tamoxifen were dissolved in DMSO, resulting in stock concentrations of 120 mM for empagliflozin and 8 mM for tamoxifen. These stock solutions were then used to prepare a dilution series in complete medium, starting from 600 μM for empagliflozin and 40 μM for tamoxifen, ensuring that the final DMSO concentration in this dilution series did not exceed 0.5%. Each experiment was conducted for a minimum of 90 h and replicated three times. Data analysis was performed using the integrated software provided by xCELLigence for calculations.

### Protein extraction and western blot analysis

MCF-7 cells were seeded into six‐well plates at a density of 1 × 10^6 cells per well. Following a 24-h incubation period, the cells were treated with empagliflozin (180 μM), tamoxifen (17.5 μM), or a combination of both (180 μM /17.5 μM), and incubated for various durations: 30 min, 1 h, 3 h, 9 h, 24 h, or 36 h. Subsequently, the cells were harvested in RIPA lysis buffer supplemented with phosphatase and protease inhibitor cocktails. Protein concentrations were determined using the BCA Protein Assay Kit (ABP Biosciences, Maryland, USA). Samples were subjected to electrophoresis on 10% SDS‒PAGE gels. The proteins were subsequently transferred onto PVDF membranes (Nepenthe, Türkiye). Nonspecific binding sites on the membranes were blocked with TBS-T containing 5% skim milk. Following blocking, the membranes were incubated overnight at 4°C with the following primary antibodies: phospho Thr172‒AMPKα (#2535; Cell Signaling Technology), phospho Ser371‒p70S6K1 (#9208, Cell Signaling Technology), phospho Thr180/Tyr182‒p38 MAPKα (#E-AB-21027, Elabscience), phospho Ser473‒Akt (#FNab06402, Fine Biotech), and GAPDH (#E-AB-40337, Elabscience). After washing, the membranes were incubated with either goat anti-rabbit IgG secondary antibodies conjugated with horseradish peroxidase (#A53211, AFG Bioscience) or goat anti-mouse IgG secondary antibodies conjugated with horseradish peroxidase (#FNSA-0003, Fine Biotech) in TBS-T for 3h at 22°C. The experiments were conducted in triplicate, and signals were detected using an enhanced chemiluminescence (ECL) kit (#SM801-0500, GeneDireX Inc., Taiwan) and the ChemiDoc XRS Imaging System. The bands were analyzed using ImageJ software (National Institutes of Health, USA).

### RNA extraction and quantitative polymerase chain reaction (qPCR)

MCF-7 cells were seeded in six-well plates at a density of 1 × 10^6 cells per well. After a 24-h incubation period, the cells were treated with empagliflozin (180 μM), tamoxifen (17.5 μM), or a combination of both (180 μM /17.5 μM) and incubated for 9 h, 24 h, or 36 h. Subsequently, mRNA was extracted using RNAzol® RT reagent (# R4533, Sigma-Aldrich) according to the manufacturer’s instructions. The quantity and purity of the RNA were assessed with a UV/Vis Nano Spectrophotometer (MicroDigital Co., Ltd., Gyeonggi-do, Korea). Next, the mRNA (1 µg) was reverse transcribed to complementary DNA (cDNA) using a WizScript™ cDNA Synthesis Kit (#W2211, Wizbiosolutions Inc.) according to the manufacturer's protocol. For cDNA quantification, qPCR was conducted using the A.B.T.™ SNPtyping Taqman assay kit for FOXO3a (#َ Q15-01–01, Atlas Biotechnology Laboratory, Ankara, Türkiye) and the A.B.T.™ 2X qPCR SYBR-Green MasterMix assay kit for PGC1α (#Q03-01–01, Atlas Biotechnology Laboratory, Ankara, Türkiye). β-actin was utilized as an internal control. The Fold change was calculated using the 2^−ΔΔCt^ method. The experiments were performed in triplicate.

### Statistical analysis

The data are presented as the mean ± standard deviation (SD). Statistical analysis was conducted using unpaired two-tailed Student’s t-test, as well as one-way analysis of variance (ANOVA), followed by Bonferroni post-test. Significance was determined at the level of *P* < 0.05. All statistical analyses were performed using GraphPad Instat software (GraphPad Software Inc., San Diego, CA, USA).

## Results

### Empagliflozin demonstrates cytotoxic effects and exhibits synergism with tamoxifen in MCF-7 breast cancer cells

**Fig.1:**
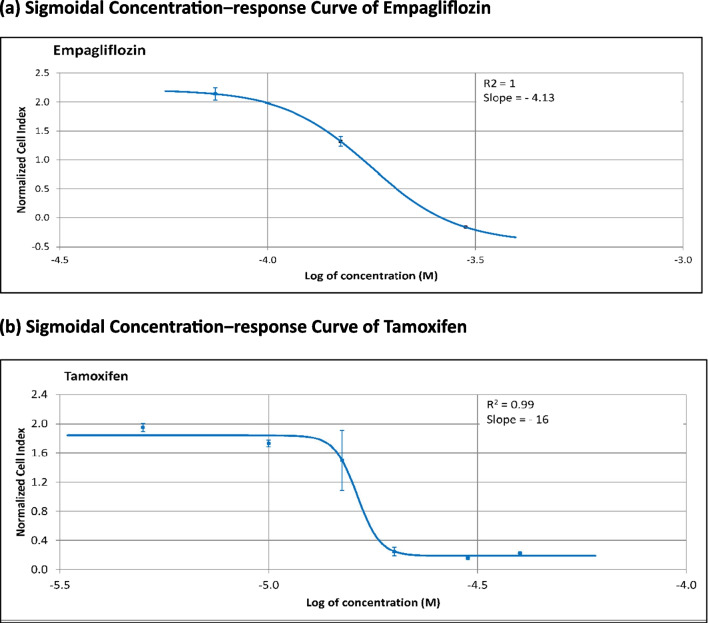
Sigmoidal concentration‒response curves of MCF-7 breast cancer cells treated with empagliflozin and tamoxifen for 48 h. Cells were exposed to different concentrations of empagliflozin (ranging from 75 to 600 μM) or tamoxifen (5 to 40 μM). Both empagliflozin and tamoxifen demonstrated concentration-dependent cytotoxic effects. The curves were automatically generated using the RTCA-integrated software of the xCELLigence system. The data are presented as the means ± standard deviatiosn (SDs), and each experiment was performed in triplicate (*n* = 3). **a** Sigmoidal Concentration‒response Curve of Empagliflozin. **b** Sigmoidal Concentration‒response Curve of Tamoxifen

**Fig. 2:**
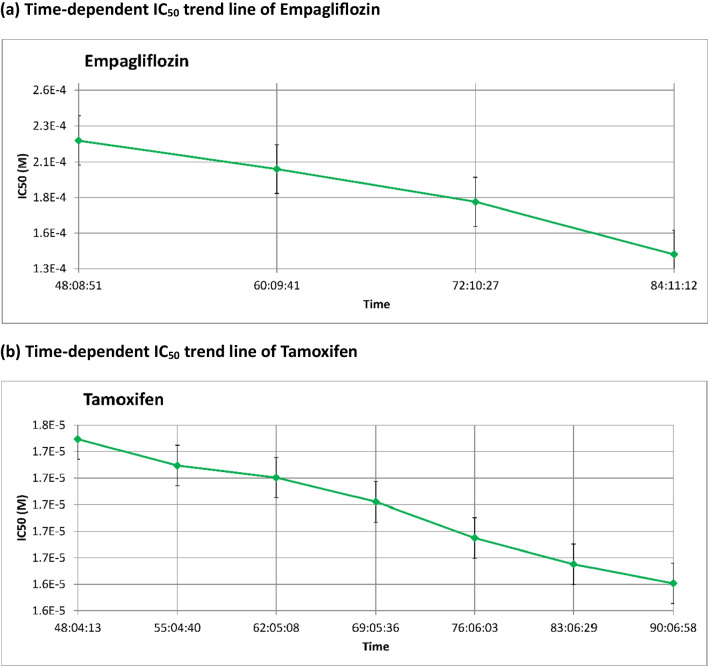
Time-dependent IC_50_ values of empagliflozin and tamoxifen in MCF-7 breast cancer cells, shown in molar concentrations (M) and scaled by factors of 10^^−4^ (E-4) and 10^^−5^ (E-5). The data are presented as the means ± standard deviations (SDs), and each experiment was conducted in triplicate (*n* = 3). **a** Time-dependent IC_50_ trend line of Empagliflozin. **b** Time-dependent IC_50_ trend line of Tamoxifen

Initially, we assessed the individual cytotoxic effects of empagliflozin and tamoxifen in MCF-7 breast cancer cells using the xCELLigence system. Subsequently, we determined the IC_50_ values of both drugs following 48 h of exposure using the integrated software of the xCELLigence system. Figure 1 illustrates the concentration-dependent cytotoxicity of each drug, revealing tamoxifen to be approximately tenfold more potent (IC_50_ = 17 μM) than empagliflozin (IC_50_ = 177 μM). Furthermore, tamoxifen exhibited a steeper sigmoidal curve compared to empagliflozin, indicating greater sensitivity to changes in concentration. Additionally, the IC_50_ values of both empagliflozin and tamoxifen decrease over time, suggesting time-dependent cytotoxicity (see Fig. 2).

**Fig. 3:**
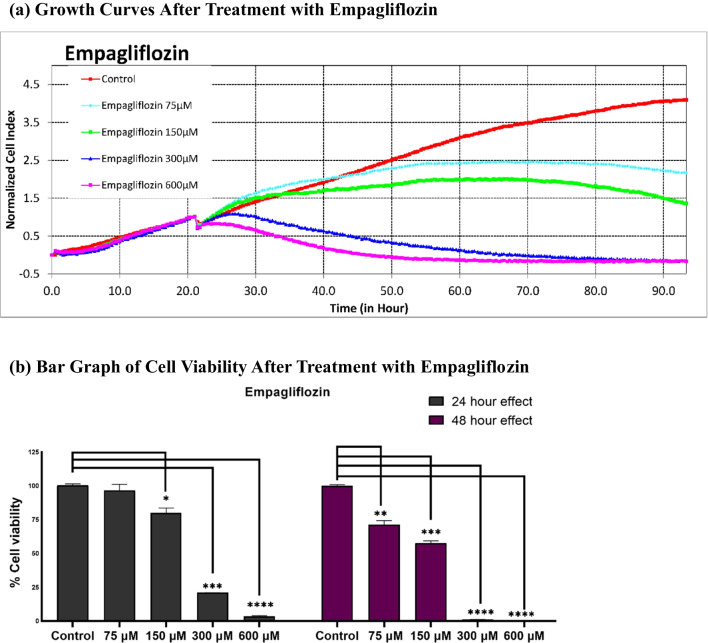

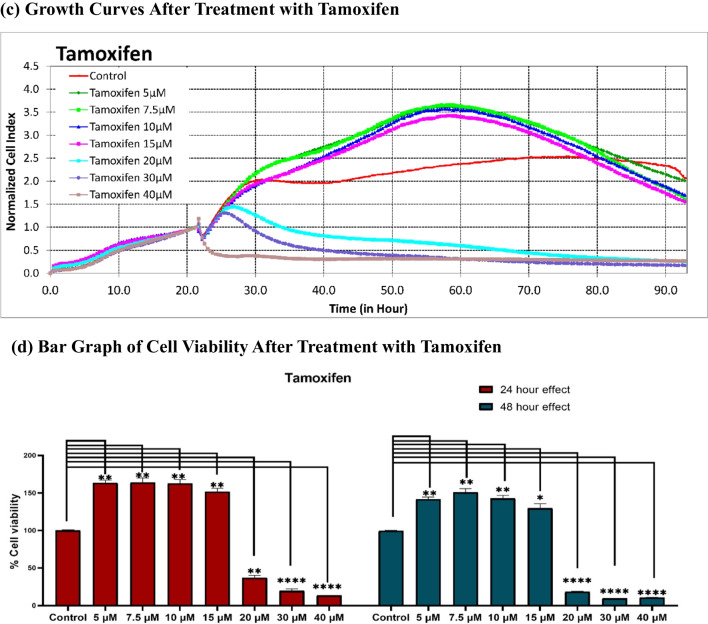
Real-time analysis of the cytotoxic effects of empagliflozin and tamoxifen using the xCELLigence System. Figures a and c illustrate the normalized cell indices of MCF-7 breast cancer cells treated with varying concentrations of empagliflozin (75–600 μM) and tamoxifen (5–40 μM), with the cell indices normalized at the time of drug administration. Figures b and d represent bar graphs of cell viability after 24 h and 48 h of treatment with empagliflozin and tamoxifen in MCF-7 breast cancer cells. Cell viability is expressed as a percentage of the mean of the untreated control. The data are presented as the means ± standard deviations (SDs) (*n* = 3). Statistical significance levels are indicated as * *p* < 0.05, ** *p* < 0.01, *** *p* < 0.001, and **** *p* < 0.0001 when compared with the control. **a** Growth Curves After Treatment with Empagliflozin. **b** Bar Graph of Cell Viability After Treatment with Empagliflozin. **c** Growth Curves After Treatment with Tamoxifen. **d** Bar Graph of Cell Viability After Treatment with Tamoxifen

Significant cytotoxicity was noted at concentrations of 75 μM for empagliflozin and 20 μM for tamoxifen, as depicted in Fig. 3. Tamoxifen at concentrations of ≥ 20 μM significantly reduced cell viability compared to that of the untreated controls. However, at concentrations less than 20 μM, the opposite effect was observed, indicating a potential biphasic or intricate response to varying concentrations. In contrast, empagliflozin demonstrated cytotoxic effects at all concentrations examined in the study.

**Fig. 4:**
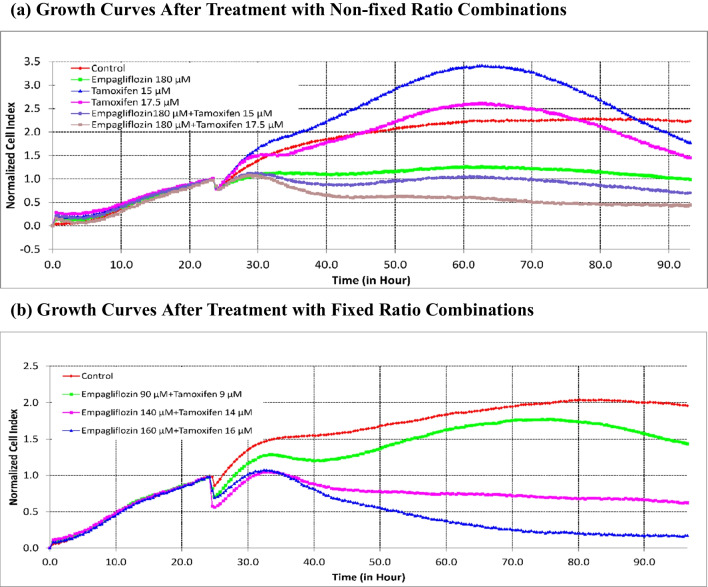

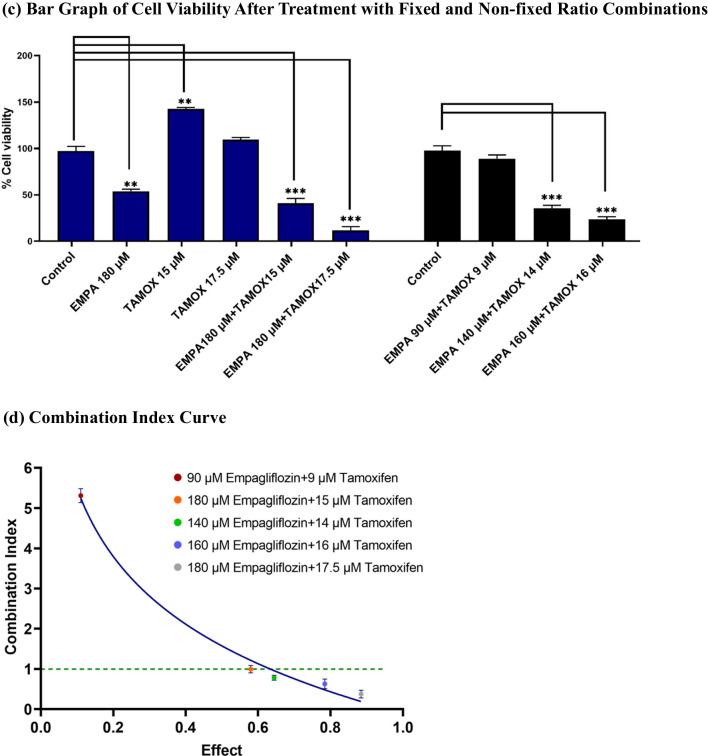
Synergistic cytotoxicity of empagliflozin and tamoxifen across multiple combination regimens. In figure a, MCF-7 breast cancer cells were treated either alone or concurrently with a constant concentration of empagliflozin (180 μM) and varying concentrations of tamoxifen (15 μM, 17.5 μM). In figure b, the cells were treated with a combination of both drugs at an equipotent concentration ratio of 10:1 (90 μM empagliflozin + 9 μM tamoxifen, 140 μM empagliflozin + 14 μM tamoxifen, 160 μM empagliflozin + 16 μM tamoxifen). Cell viability was evaluated using the xCELLigence System, and the cell indices were normalized at the time of drug administration. Figure c shows a bar graph depicting cell viability after 48 h of treatment with singly or concurrently administered empagliflozin and tamoxifen to MCF-7 breast cancer cells. Viability is expressed as a percentage of the mean of the untreated control. The data are presented as the means ± standard deviation (SDs) (*n* = 3), with statistical significance levels indicated as * *p* < 0.05, ** *p* < 0.01, and *** *p* < 0.001 when compared with the control. Figure d illustrates the combination index (CI) plot of the different combination regimens. Values falling below and above the dashed line (CI = 1) indicate synergism and antagonism, respectively. The data are presented as the means ± standard deviations (SDs) (*n* = 3). **a** Growth Curves After Treatment with Non-fixed Ratio Combinations. **b** Growth Curves After Treatment with Fixed Ratio Combinations. **c** Bar Graph of Cell Viability After Treatment with Fixed and Non-fixed Ratio Combinations. **d** Combination Index Curve

Our subsequent investigation aimed to determine whether empagliflozin could exhibit synergistic effects when combined with tamoxifen. MCF-7 breast cancer cells were subjected to a combination of a constant concentration of empagliflozin and varying concentrations of tamoxifen, particularly near the IC_50_, given the heightened sensitivity of tamoxifen to concentration fluctuations (see Fig. 4a and c). Furthermore, cells were exposed to a combination of empagliflozin and tamoxifen at an equipotent concentration ratio of 10:1, considering tamoxifen's approximately tenfold greater potency than empagliflozin (see Fig. 4b and c). To assess synergistic effects, the combination index (CI) values were determined with CompuSyn software using the Chou-Talalay method (Chou 2010), where CI values < 1, = 1, or > 1 denote synergistic, additive, or antagonistic activity, respectively (see Fig. 4d).

Synergistic cytotoxicity was observed for several combinations in which tamoxifen concentrations (14 μM, 15 μM, 16 μM, and 17.5 μM) were near IC_50_ (17 μM). The combined effect of empagliflozin and tamoxifen on MCF-7 breast cancer cells appeared to diminish as the combination concentrations deviated further from the IC_50_ of both drugs, and it may even transition into antagonism at concentrations near half of the IC_50_ for both drugs (refer to Fig. 4d).

We also computed DRI (dose-reduction index) values using CompuSyn software. The DRI represents the actual fold change in dose attenuation resulting from a synergistic combination at a given effect level compared with that resulting from the drug alone. Table 1 demonstrates that the DRIs of empagliflozin and tamoxifen are greater than 1, indicating favorable dose reduction when these agents are combined (Chou 2010).

**Table 1:** Dose reduction index (DRI) of empagliflozin and tamoxifen. Values were calculated using CompuSyn software and presented as the means ± standard deviations (SDs) (*n* = 3)

Combination	Dose reduction index (DRI)	
	Empagliflozin	Tamoxifen
180 μM Empagliflozin + 15 μM Tamoxifen	1.69 ± 0.35	2.39 ± 0.05
180 μM Empagliflozin + 17.5 μM Tamoxifen	11.34 ± 1.50	2.46 ± 0.03
90 μM Empagliflozin + 9 μM Tamoxifen	0.24 ± 0.06	3.11 ± 0.07
140 μM Empagliflozin + 14 μM Tamoxifen	3.23 ± 0.29	2.67 ± 0.02
160 μM Empagliflozin + 16 μM Tamoxifen	5.97 ± 0.11	2.50 ± 0.01

Based on the individual and combined cytotoxic effects, we utilized the combination of 180 μM empagliflozin and 17.5 μM tamoxifen for subsequent assays.

### Empagliflozin upregulates the activity of AMPKα and downregulates the activity of Akt, p70S6K1, and p38 MAPKα

The activities of AMPKα, Akt, p70S6K1, and p38 MAPKα were evaluated using Western blotting.

**Fig. 5:**
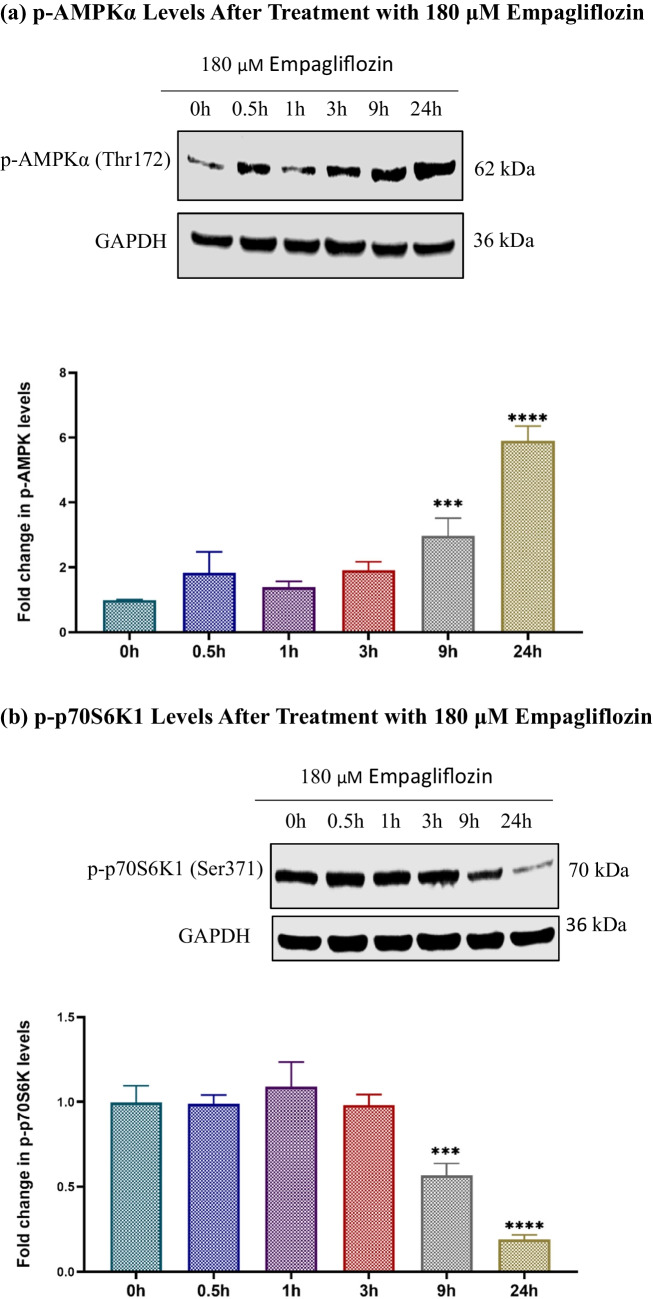

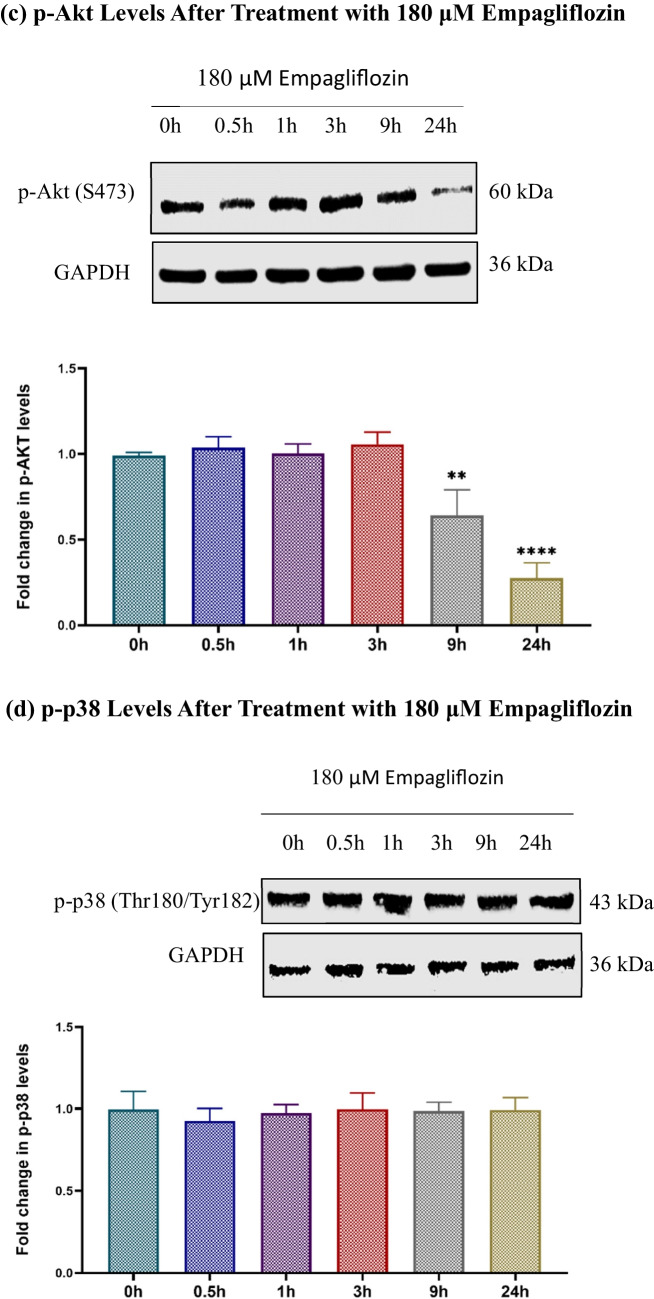
Immunoblots depict the levels of p-AMPKα (a), p-p70S6K1 (b), p-Akt (c), and p-p38 MAPKα (d) in the lysates of MCF-7 breast cancer cells treated with 180 μM empagliflozin for up to 24 h. Quantitative normalization was conducted using GAPDH as an internal control. The values are expressed as fold change relative to untreated control cells. The data are presented as the means ± standard deviations (SDs) (*n* = 3). Statistical significance levels are indicated as ** *p* < 0.01, *** *p* < 0.001, **** *p* < 0.0001 compared to the control. **a** p-AMPKα Levels After Treatment with 180 μM Empagliflozin. **b** p-p70S6K1 Levels After Treatment with 180 μM Empagliflozin. **c** p-Akt Levels After Treatment with 180 μM Empagliflozin. **d** p-p38 Levels After Treatment with 180 μM Empagliflozin

Initially, we examined the individual effects of empagliflozin and determined the optimal time points. Subsequently, we investigated the combined effect of these two agents. Treatment with empagliflozin led to a significant increase in p-AMPKα levels for up to 24 h, along with a consistent decrease in p-p70S6K1 and p-Akt levels (Fig. 5). However, empagliflozin showed no significant impact on p-p38 MAPKα levels.

**Fig. 6:**
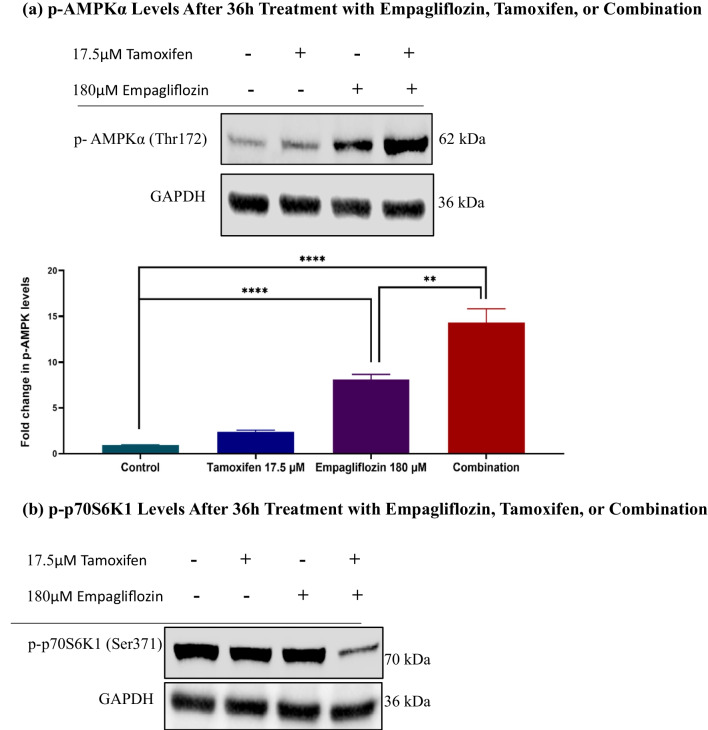

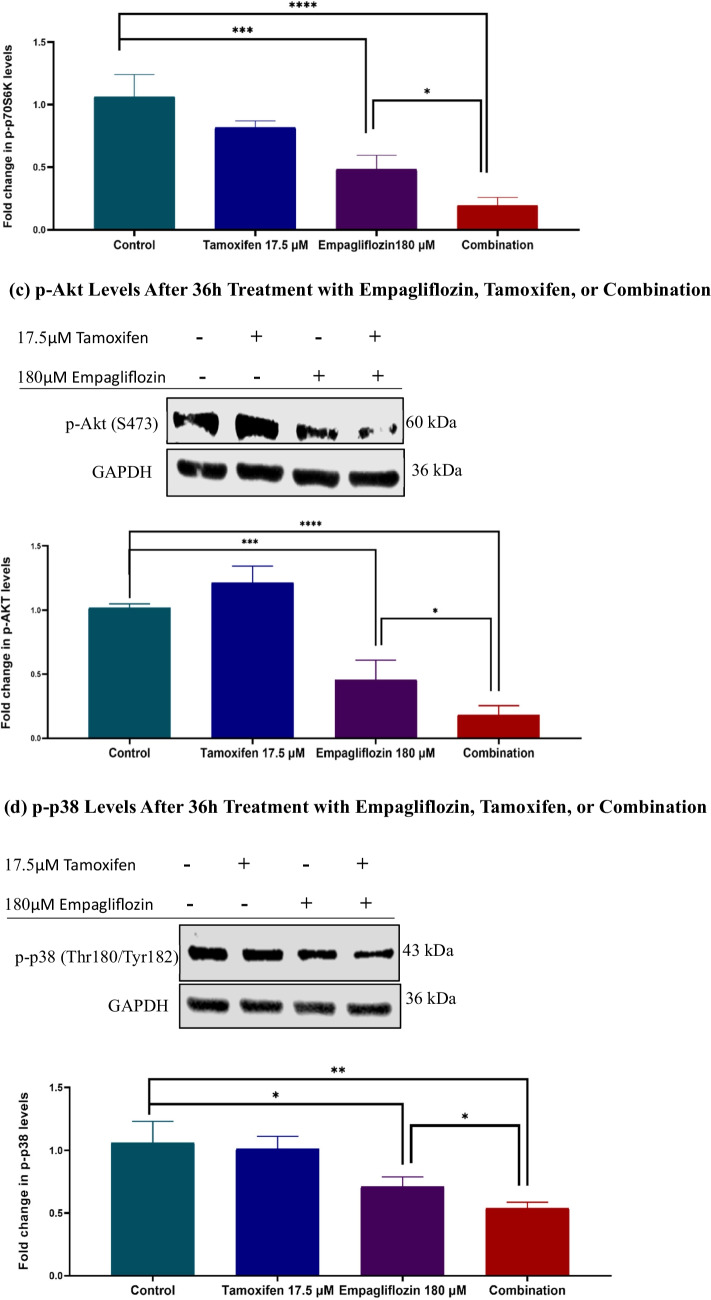
Immunoblots showing the levels of p-AMPKα (a), p-p70S6K (b), p-Akt (c), and p-p38 MAPKα (d) after 36h of treating MCF-7 breast cancer cells with 180 μM empagliflozin, 17.5 μM tamoxifen, or a combination of both drugs. Quantitative normalization was performed using GAPDH as an internal control. The values are presented as the fold change relative to untreated control cells. The data are presented as the means ± standard deviations (SDs) (*n* = 3). Statistical significance is indicated as * *p* < 0.05, ** *p* < 0.01, *** *p* < 0.001, **** *p* < 0.0001 compared to the control or empagliflozin. **a** p-AMPKα Levels After 36h Treatment with Empagliflozin, Tamoxifen, or Combination. **b** p-p70S6K1 Levels After 36h Treatment with Empagliflozin, Tamoxifen, or Combination. **c** p-Akt Levels After 36h Treatment with Empagliflozin, Tamoxifen, or Combination. **d** p-p38 Levels After 36h Treatment with Empagliflozin, Tamoxifen, or Combination

Upon combining empagliflozin with tamoxifen, these effects were significantly enhanced at 36 h postexposure, despite the individual effect of tamoxifen not reaching significance (Fig. 6).

Building on the observed effects of empagliflozin and its combination with tamoxifen on intracellular signaling pathways (AMPKα, Akt, p70S6K1, and p38 MAPKα), the study examined their impact on gene expression levels of PGC-1α and FOXO3a.

### Empagliflozin decreases the gene expression of PGC-1α and increases the gene expression of FOXO3a

The gene expression levels of PGC-1α and FOXO3a were assessed via q-PCR.

**Fig. 7:**
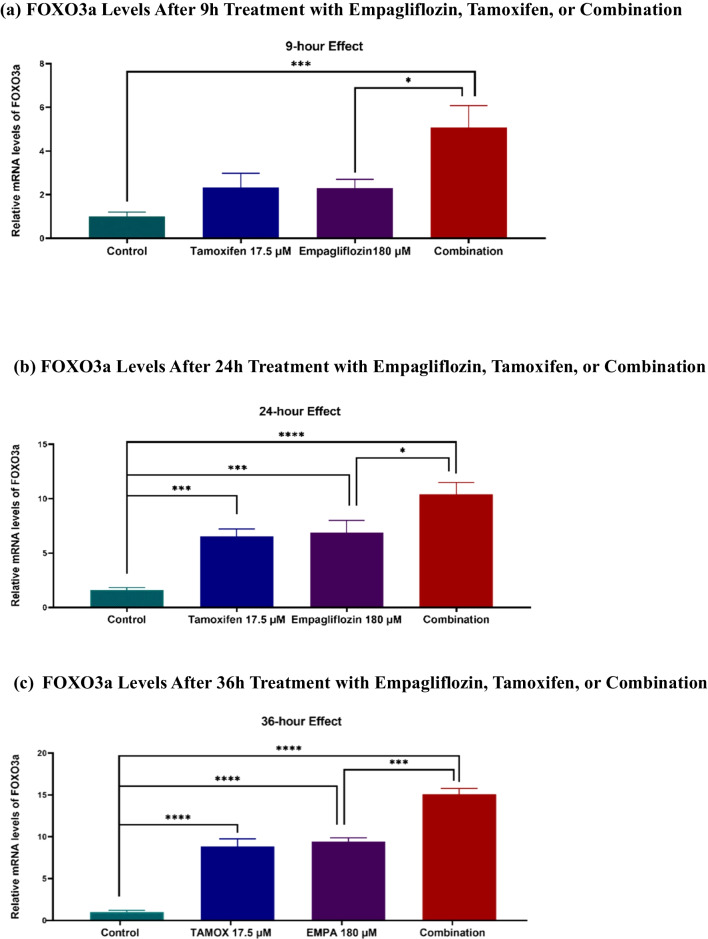

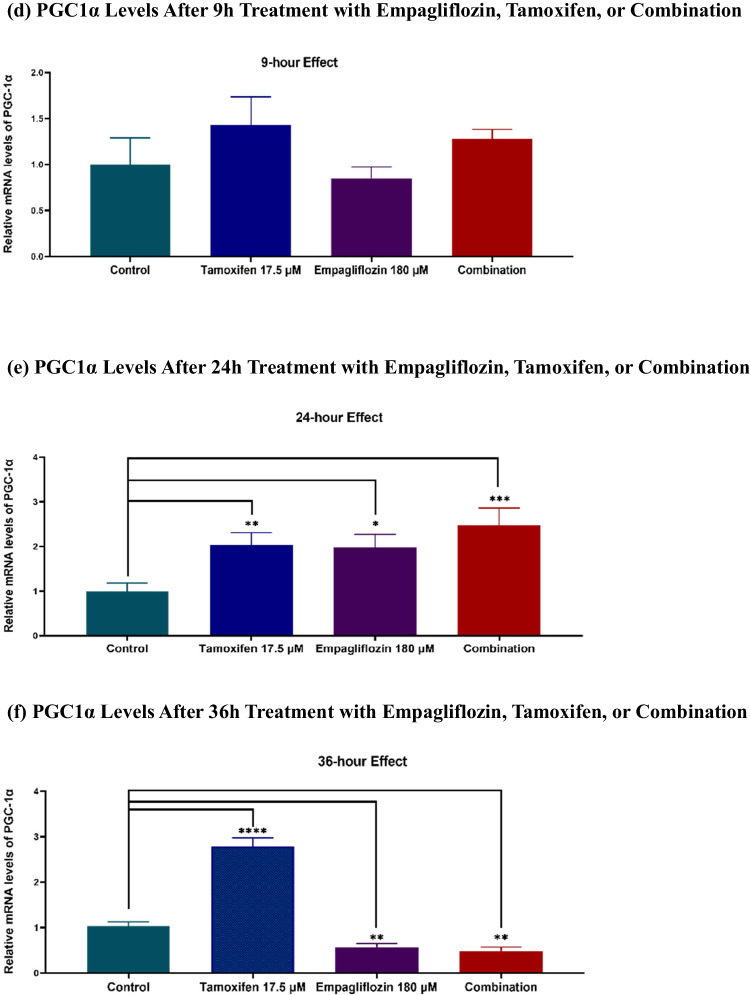
Relative mRNA levels of FOXO3a (a-c) and PGC1α (d-f) at various time points (9h, 24h, 36h) following treatment of MCF-7 breast cancer cells with empagliflozin at 180 μM, tamoxifen at 17.5 μM, or a combination of both drugs. Quantitative normalization was performed using β-actin as an internal control. The data represent fold changes relative to untreated control cells and are presented as the means ± standard deviations (SDs) (*n* = 3). Statistical significance is denoted as * *p* < 0.05, ** *p* < 0.01, *** *p* < 0.001, **** *p* < 0.0001 compared with the control or empagliflozin. **a** FOXO3a Levels After 9h Treatment with Empagliflozin, Tamoxifen, or Combination. **b** FOXO3a Levels After 24h Treatment with Empagliflozin, Tamoxifen, or Combination. **c** FOXO3a Levels After 36h Treatment with Empagliflozin, Tamoxifen, or Combination. **d** PGC1α Levels After 9h Treatment with Empagliflozin, Tamoxifen, or Combination. **e** PGC1α Levels After 24h Treatment with Empagliflozin, Tamoxifen, or Combination. **f** PGC1α Levels After 36h Treatment with Empagliflozin, Tamoxifen, or Combination

Over a 36-h period, there was a statistically significant increase in the gene expression of FOXO3a, corresponding with the observed elevation in AMPK levels following empagliflozin treatment as well as the combination treatment (see Fig. 7). In contrast, although the gene expression of FOXO3a was significantly increased in tamoxifen-treated cells, this upregulation did not correlate with the fluctuations in AMPK levels. This incongruence suggests a close association between FOXO3a activity and AMPK levels.

Like in MCF-7 breast cancer cells treated with empagliflozin or tamoxifen alone, in combination-treated cells, PGC-1α levels were significantly increased at 9h and 24h postexposure (Fig. 7). However, at 36h postexposure, this elevation significantly decreased to a level comparable to that observed in empagliflozin-treated cells, unlike that in the cells treated with tamoxifen, which demonstrated a 2.8-fold increase compared to that in the control. This observation suggests that the effect of the combination treatment may be primarily driven by empagliflozin, which manifested its effect after 36 h of exposure.

## Discussion

This study investigated empagliflozin's anticancer effects and its synergy with tamoxifen in MCF-7 breast cancer cells. Empagliflozin has been shown to demonstrate anticancer effects in vitro at concentrations ranging from 75 μM to 600 μM, yet clinical dosing in humans yields markedly lower serum concentrations, ranging from 0.259 μM to 2.39 μM (Heise et al. 2013). A crude estimate suggests that a dose of 3138 mg/day is needed to achieve a serum concentration of 75 μM. However, using such high doses poses challenges for oncological use due to safety concerns not addressed in clinical trials. Hence, further studies are vital to assess the safety and efficacy of empagliflozin for anticancer therapy at these doses. Additionally, employing new technologies to target empagliflozin accumulation within tumors could enhance its anticancer effectiveness.

Utilizing the synergistic effects of drugs used in clinical oncology could be pivotal for the future safe and effective utilization of empagliflozin in oncological contexts. Exploring the synergy between empagliflozin and tamoxifen, we found that their concurrent administration, near their respective IC_50_ values, led to substantial cell death (64%–88%). Notably, the dose-reduction indices reached 11.34 for empagliflozin and 3.11 for tamoxifen, indicating potential dosage optimization for both agents. However, further studies are needed to determine the ideal combination ratio and regimen (e.g., simultaneous, or sequential treatment) for maximizing the synergistic effects on MCF-7 breast cancer cells.

Our findings indicate that empagliflozin activates AMPKα in a time-dependent manner, consistent with increase in cytotoxicity. Despite the high metabolic demands of tumors, AMPKα is not phosphorylated in the majority of primary human breast cancers, suggesting dysfunction in its activation process. Brown et al. (2011) showed that estradiol-induced downregulation of LKB1 decreased AMPKα phosphorylation. While our study did not reveal the exact mechanism of AMPKα activation, our results align with those reported for empagliflozin in other cancer cell types (Abdelhamid et al. 2022; Xie et al. 2020).

In the context of endocrine therapy resistance, several endocrine therapy resistance mechanisms have been reported to repress AMPKα (Casimiro et al. 2017; Lopez-Mejia et al. 2017; Yi et al. 2020). Evidence for AMPKα repression's role in endocrine therapy resistance was demonstrated in luminal breast cancer cell line models resistant to tamoxifen, in which increased repression of AMPKα was observed in resistant cells compared to sensitive cells. Additionally, drugs activating AMPKα have been shown to inhibit the growth of endocrine therapy resistant breast cancer (Berstein et al. 2011). Therefore, empagliflozin and the combination may potentially restore sensitivity to tamoxifen, warranting further investigation to confirm this effect.

Our study assessed p-p70S6K1, p-Akt, and FOXO3a expression levels to confirm the anticancer effects of empagliflozin and its potential to combat tamoxifen resistance. Both empagliflozin and the combination treatment notably decreased p-p70S6K1 expression, which was correlated with increased p-AMPKα levels. This decrease is vital because p70S6K functions as a downstream effector of the PI3K/Akt/mTOR pathway, which is frequently upregulated in cases of breast cancer (Bärlund et al. 2000). The mechanism of action of empagliflozin appears to rely on AMPKα activation, which prevents mTORC1 from modulating p70S6K, and other proteins involved in protein synthesis.

Elevated p70S6K expression is common in cancer cell lines resistant to various chemotherapeutic drugs and is associated with endocrine resistance and poor prognosis in hormone receptor-positive breast cancers (Kim et al. 2011). Moreover, nuclear accumulation of p70S6K has been linked to a reduced benefit from tamoxifen treatment (Bostner et al. 2015).

Another downstream target of AMPKα is FOXO3a, which is directly phosphorylated by AMPKα to enhance its transcriptional activity. FOXO3a serves as a tumor suppressor in breast cancer by increasing the expression of the pro-apoptotic protein BIM (Arden 2006; Greer et al. 2007). Studies suggest that re-expression of FOXO3a can restore sensitivity to tamoxifen and reduce tumor mass in tamoxifen-resistant mouse models (Pellegrino et al. 2019; Ricci et al. 2022). Both empagliflozin and the combination treatment significantly increased FOXO3a gene expression and correlated with increased p-AMPKα and decreased p-Akt levels. However, while FOXO3a gene expression increased significantly in tamoxifen-treated cells, this change did not correlate with p-AMPKα levels. This finding suggests a close association between FOXO3a activity and AMPKα activity. Although this study did not directly demonstrate FOXO3a activity, it provides a basis for future investigations by revealing the transcriptional upregulation of FOXO3a mRNA levels in response to empagliflozin and combination treatment.

PTEN has been identified as a target of FOXO3a, and its low expression has been documented in ER + breast cancer. FOXO3a enhances PTEN transcription to counteract PI3K/Akt pathway hyperactivity (Nasimian et al. 2020; Sajjadi et al. 2021). Our study showed that empagliflozin and the combination treatment decreased Akt activity in line with the increase in AMPKα activity and FOXO3a gene expression. However, further research is needed to determine whether empagliflozin and the combination treatment exclusively target the FOXO3a/PTEN pathway or involve other pathways. Nevertheless, this finding is in consistent with previous findings by Abdelhamid et al (2022). In ER + breast cancer, the interplay between PI3K-Akt-mTOR pathway and estrogen receptor α pathway drives resistance to endocrine therapy (Cassinelli et al. 2013), suggesting that combination therapy with tamoxifen and empagliflozin could prevent or overcome resistance to anti-hormonal therapy.

Despite numerous studies highlighting AMPKα's anti-cancer role, AMPKα can promote tumor survival by adjusting cellular metabolism to maintain energy balance. It upregulates PGC-1α, enhancing mitochondrial metabolism and biogenesis, along with other metabolic processes crucial for cancer cell survival (Chaube et al. 2015; Leone et al. 2005; Michael et al. 2001; Valle et al. 2005). Interestingly, the AMPKα-p38 MAPKα-PGC-1α axis supports cancer cell survival during glucose limitation (Chaube et al. 2015). Our study demonstrated that both empagliflozin and the combination treatment significantly affect p-p38 MAPKα levels, consistent with previous findings indicating that empagliflozin inhibits p38 MAPKα (Abdelhamid et al. 2022).

Overexpression of PGC-1α and its target glutaminolysis genes are associated with poor prognosis in breast cancer patients, and high PGC-1α expression has been found in circulating cancer cells, supporting invasiveness in a mouse model of breast cancer (LeBleu et al. 2014; McGuirk et al. 2013). Our study showed that empagliflozin and the combination treatment significantly decreased the gene expression of PGC-1α after 36 h of administration. These findings coincide with the decrease in p38 MAPKα activity at 36 h postexposure to empagliflozin and the combination treatment, suggesting a potential role for empagliflozin in preventing metastasis. Nonetheless, further preclinical and clinical studies are essential to confirm the antimetastatic effects of empagliflozin.

While our study provides important insights into the anticancer effects of empagliflozin and its synergistic effects with tamoxifen in the MCF-7 ER + breast cancer cell line, it is important to acknowledge that the use of a single cell line may not fully represent the heterogeneity of ER + breast cancers. Future studies should aim to replicate our findings in additional ER + breast cancer cell lines, such as T47D and ZR-75–1, to ensure the robustness and generalizability of the results.

## Conclusion

This study highlights the anticancer effects of empagliflozin in MCF-7 breast cancer cells, both individually and in synergy with tamoxifen. Empagliflozin exerts anti-proliferative and anti-survival effects by inhibiting mTOR, Akt, and PGC-1α and it exhibits synergy with tamoxifen in MCF-7 cells. Overall, this study sets the stage for further exploration of the anticancer potential of empagliflozin in ERα + breast cancer.

## Supplementary Information

Below is the link to the electronic supplementary material.

Supplementary file1 (PDF 176 KB)

## Data Availability

No datasets were generated or analysed during the current study.

## Abbreviations

AMPKα: AMP-activated protein kinase α
CI: Combination index
DRI: Dose reduction index
ER: Estrogen receptor
FOXO3a: Forkhead box O3a
PGC-1α: Peroxisome proliferator-activated receptor-gamma coactivator-1α
PTEN: Phosphatase and tensin homolog
p38 MAPKα: P38 mitogen-activated protein kinase
p70S6K1: P70-S6 kinase 1
SGLT2: Sodium-glucose cotransporter 2
