# Supplementary figures and images for: Empagliflozin demonstrates cytotoxicity and synergy with tamoxifen in ER-positive breast cancer cells: anti-proliferative and anti-survival effects
Source: Naunyn Schmiedebergs Arch Pharmacol. 2024 Jul 27;398(1):781–98. doi: 10.1007/s00210-024-03316-z (PMC11787280; doi:10.1007/s00210-024-03316-z)

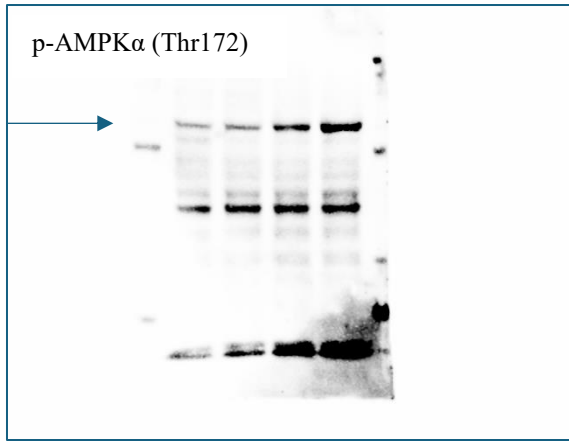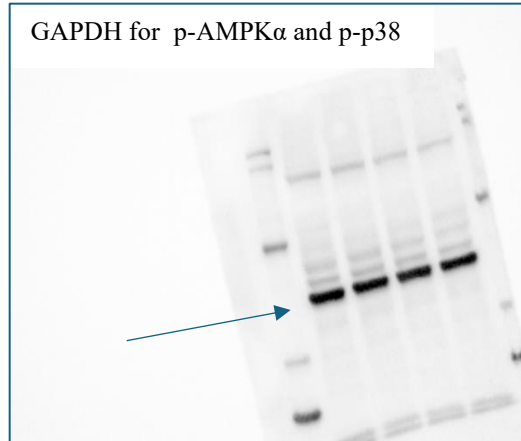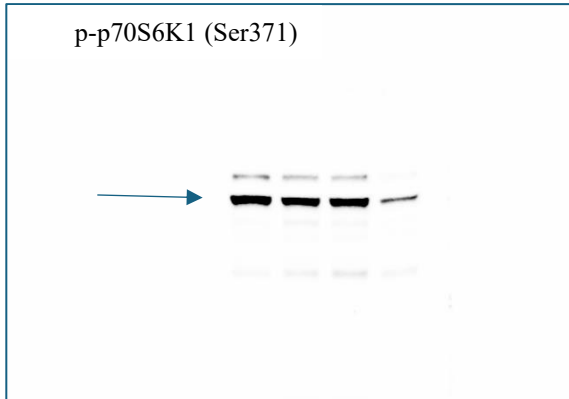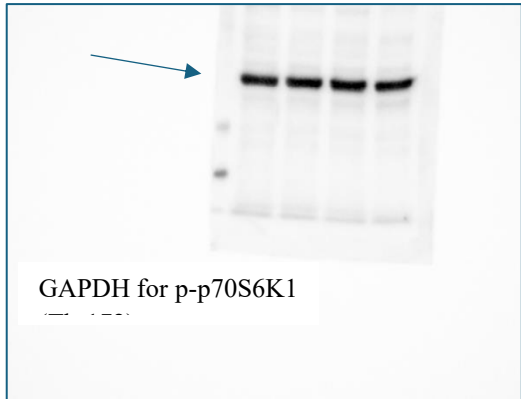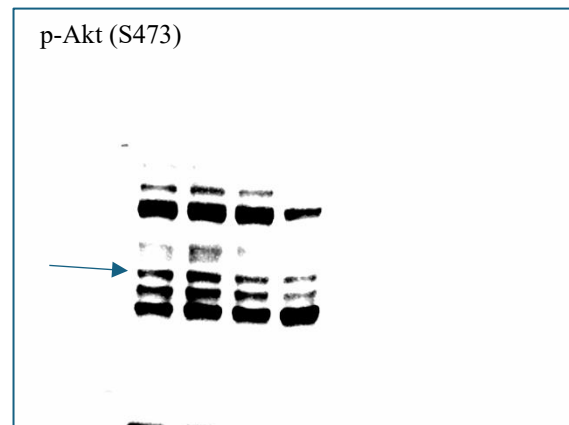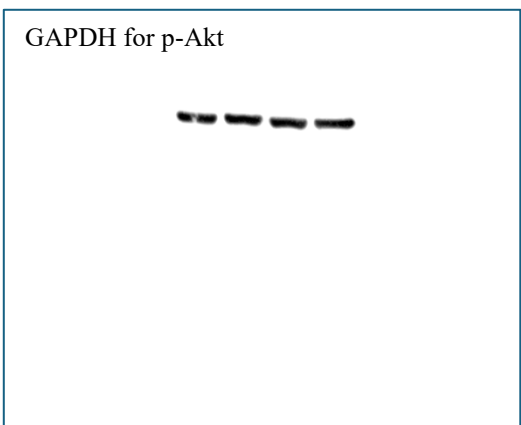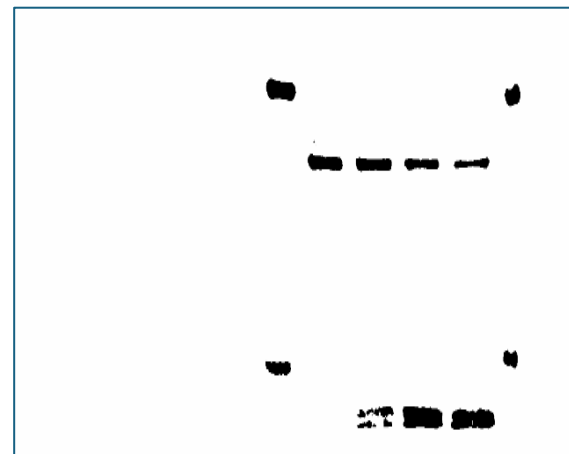

p-AMPK $\alpha$  (Thr172)

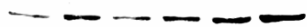

GAPDH for p-AMPK $\alpha$

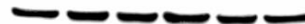

p-p70S6K1 (Ser371)

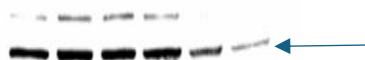

GAPDH

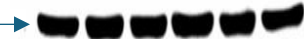

p-Akt (S473)

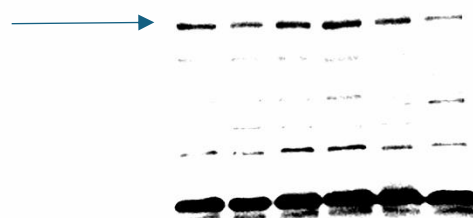

p-p38 (Thr180/Tyr182)

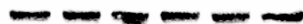

GAPDH for p-Akt

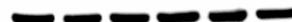

GAPDH for p-p38

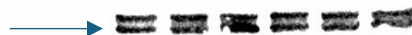

Supplement: Supplementary file 1 — Supplementary file1 (PDF 176 KB) [file 210_2024_3316_MOESM1_ESM.pdf]
